# Effective Range of FSSW Parameters for High Load-Carrying Capacity of Dissimilar Steel A283M-C/Brass CuZn40 Joints

**DOI:** 10.3390/ma15041394

**Published:** 2022-02-14

**Authors:** Sabbah Ataya, Mohamed M. Z. Ahmed, Mohamed M. El-Sayed Seleman, Khalil Hajlaoui, Fahamsyah H. Latief, Ahmed M. Soliman, Yousef G. Y. Elshaghoul, Mohamed I. A. Habba

**Affiliations:** 1 Department of Mechanical Engineering, College of Engineering, Imam Mohammad Ibn Saud Islamic University, Riyadh 11432, Saudi Arabia; smataya@imamu.edu.sa (S.A.); kmhajlaoui@imamu.edu.sa (K.H.); fhlatief@imamu.edu.sa (F.H.L.); 2 Department of Metallurgical and Materials Engineering, Faculty of Petroleum and Mining Engineering, Suez University, Suez 43512, Egypt; mohamed.elnagar@suezuniv.edu.eg; 3 Mechanical Engineering Department, College of Engineering at Al Kharj, Prince Sattam Bin Abdulaziz University, Al Kharj 16273, Saudi Arabia; 4 Department of Mechanical Engineering, College of Engineering, Jouf University, Sakaka 72388, Saudi Arabia; amsoliman@ju.edu.sa; 5 Mechanical Engineering Department, Faculty of Engineering, Suez University, Suez 43518, Egypt; yousef.gamal@eng.suezuniv.edu.eg; 6 Mechanical Department, Faculty of Technology & Education, Suez University, Suez 43518, Egypt; mohamed.atia@suezuniv.edu.eg

**Keywords:** Friction stir spot welding, low-carbon steel, brass, load-carrying capacity, hardness, microstructure

## Abstract

In the current study, a 2 mm thick low-carbon steel sheet (A283M—Grade C) was joined with a brass sheet (CuZn40) of 1 mm thickness using friction stir spot welding (FSSW). Different welding parameters including rotational speeds of 1000, 1250, and 1500 rpm, and dwell times of 5, 10, 20, and 30 s were applied to explore the effective range of parameters to have FSSW joints with high load-carrying capacity. The joint quality of the friction stir spot-welded (FSSWed) dissimilar materials was evaluated via visual examination, tensile lap shear test, hardness test, and macro- and microstructural investigation using SEM. Moreover, EDS analysis was applied to examine the mixing at the interfaces of the dissimilar materials. Heat input calculation for the FSSW of steel–brass was found to be linearly proportional with the number of revolutions per spot joint, with maximum heat input obtained of 11 kJ at the number of revolutions of 500. The temperature measurement during FSSW showed agreement with the heat input dependence on the number of revolution. However, at the same revolutions of 500, it was found that the higher rotation speed of 1500 rpm resulted in higher temperature of 583 °C compared to 535 °C at rotation speed of 1000 rpm. This implies the significant effect for the rotation speed in the increase of temperature. The macro investigations of the friction stir spot-welded joints transverse sections showed sound joints at the different investigated parameters with significant joint ligament between the steel and brass. FSSW of steel/brass joints with a number of revolutions ranging between 250 to 500 revolutions per spot at appropriate tool speed range (1000–1500 rpm) produces joints with high load-carrying capacity from 4 kN to 7.5 kN. The hardness showed an increase in the carbon steel (lower sheet) with maximum of 248 HV and an increase of brass hardness at mixed interface between brass and steel with significant reduction in the stir zone hardness. Microstructural investigation of the joint zone showed mechanical mixing between steel and brass with the steel extruded from the lower sheet into the upper brass sheet.

## 1. Introduction

In 1991, friction stir welding (FSW) was initially introduced by TWI in the United Kingdom as a newly developed solid-state welding process, particularly for joining aluminum alloys with specific requirements that should be fulfilled [1,2]. Then friction stir spot welding (FSSW) was developed as one of its variant for local joining of similar and dissimilar sheets [3,4,5,6,7,8,9] as a promising technique with similarities to the basic concepts of linear FSW with a specific requirement where no lateral movement of the tool is required [10]. More interestingly, the rotating tool pierces the sheets that are being welded and then produces a stir zone. The stir zone or nugget in the friction processed materials characterized by fine, dynamic, recrystallized microstructures due the severe plastic deformation experienced at high temperatures [11,12,13,14]. Indeed, the FSSW can be operated under the bulk melting point, which allows this technique to prevent the formation of defects attributed to porosity, solidification, thermal distortion, etc. [15,16]. The FSSW technique has been applied for joining dissimilar materials [17] such as aluminum–magnesium [18], aluminum–copper [19], and aluminum–steel [10,20]. Moreover, previous investigations stated that FSSW had been employed for various steels such as high-strength [21], advanced high-strength [22], and ultrahigh-strength steels [23]. Moreover, the steels comprising mixture structures of ferrite and martensite are termed dual-phase (DP) steel. These DP steels are actually a kind of low-carbon steel consisting of ferrite and martensite microstructures. DP steels have captured particular interest due to their good combination of high strength, ductility, and weldability of these alloys and have been used widely for over the past few years in the automotive industry [24]. Some methods are used to improve the mechanical properties of DP steels: one of them is adding alloying elements into the DP steels to modify their microstructure. However, as a result of the addition of the alloying elements at a high percentage, it may cause the DP steel to become more prone to cracking during the welding process [25]. Many attempts have been carried out to join steel sheets of interstitial-free (IF) and dual-phase (DP) steel sheets by FSSW with a convex shoulder tool. Two different combinations were used: one with IF as the top sheet (IF/DP) and another with DP as the top sheet (DP/IF) [26]. Turning to copper [27] and its alloys [28], they have been widely used in many engineering applications due copper’s excellent electrical and thermal conductivities, superior corrosion resistance, and good strength and ductility. More importantly, conventional fusion welding processes cannot be undertaken to manufacture copper joints since they demonstrate high thermal diffusivity, which is approximately 10–100 times higher compared with nickel alloys and most steels [29]. Meran [30] reported that copper and copper alloys have been successfully manufactured via the FSW method. Gao et al. [31] investigated the properties of dissimilar lap joints of commercial brass (CuZn40) to plain carbon steel (S25C) by considering the welding speed effect using the FSW method. The results showed that the grain size, hardness at the stirred zone, and tensile shear fracture load of the joints were significantly changed when the welding speed were varied. With respect to brass (Cu–Zn alloy), it demonstrates high strength, plasticity, high hardness, and good corrosion resistance, which makes brass suitable to be used as structural materials in many industrial applications [32]. Nevertheless, it is very difficult to clad brass to steel by fusion welding because of the strength loss in the fusion zone due to the evaporation of Zn, as well as the large differences in the thermal physical properties between brass and steel, such as melting point, thermal conductivity, and thermal expansion coefficient [31]. To handle this problem, friction welding as a solid-state welding process has been employed in recent years. Luo et al. [33] used the CT-130-type special inertia friction welding machine to finish the H90 brass/high carbon steel dissimilar metals radial friction welding process, and Kimura et al. [34] achieved the brass/low-carbon steel dissimilar metal welding by friction welding. However, the friction welding has a large limitation in the shape of the joint, which should be a body of rotation such as a pipe and a rod-type joint. In addition, yellow brass (Cu/Zn 63/37) has an excellent capacity for cold working and tin coating by hot dipping; moreover, brass CuZn37 has excellent cold working properties [35]. Most brasses are not normally ranked as heat-treatable; some brasses are cast or hot-worked in the duplex α/β state and then annealed at about 450 °C to convert the microstructure into a single phase of better resistance to corrosion [35]. Due to the relative high temperature associated with FSSW of brass, recrystallization accompanied with microsegregation leading to separation of β-phase takes place [31], which leads to some softening. This behavior could be avoided either by applying proper stirring speed [31] or by postweld heat treatment [34].

There are some research works on the friction stir spot welding of similar and dissimilar metals and alloys [36,37,38,39]. However, limited reports are found on the friction stir spot welding of low-carbon steel and brass, which is important for the use of joints. Based on the above reasons, it is interesting to study the manufacturing of low-carbon steel and brass as a lap joint by FSSW method. The aim of this study is to investigate the effective range of FSSW combinations of parameters of rotational speed and dwell time to obtain a high load-carrying capacity of dissimilar steel A283M-C/brass CuZn40 sheet FSSW joints. The examined rotational speed are 1000, 1250, and 1500 rpm, and the applied dwell times are 5, 10, 20, and 30 s. The resulting load-carrying capacity of the joints are measured and judged by the resulting heat generated and temperature raise at the joint during the FSSW process. Moreover, the cohesion quality of the joints is examined by the metallographic examination and EDS analysis. Normalized process parameters will be determined to function in the selection of the effective range of conditions for further FSSW processes. 

## 2. Methodology 

### 2.1. Materials

The FSSW was carried out on lap joints of the brass (CuZn40) and low-carbon steel. CuZn40 brass samples were cut from cold-rolled sheet of 1 mm thickness produced by Wieland Company, Ulm, Germany. The low-carbon steel (St 44-2) sheet was produced and delivered by Ezz Dekheila Steel (EZDK) Company, Alexandria, Egypt, in the form of 2 mm thick sheets. The chemical composition of the used low-carbon steel and brass have been analyzed using Foundry-Master pro, Oxford Instruments, Abingdon, UK. The steel grade is equivalent to A283M grade C according to ASTM A283M-18 [40]. Preparing for the FSSW lap joints, the starting copper and steel sheets were cut into plates of the width of 100 mm and length of 200 mm. The chemical composition of both starting materials is listed in Table 1. The mechanical properties that were obtained according to ASTM E8/E8M-21 [41] are also included in Table 2.

**Figure 1:**
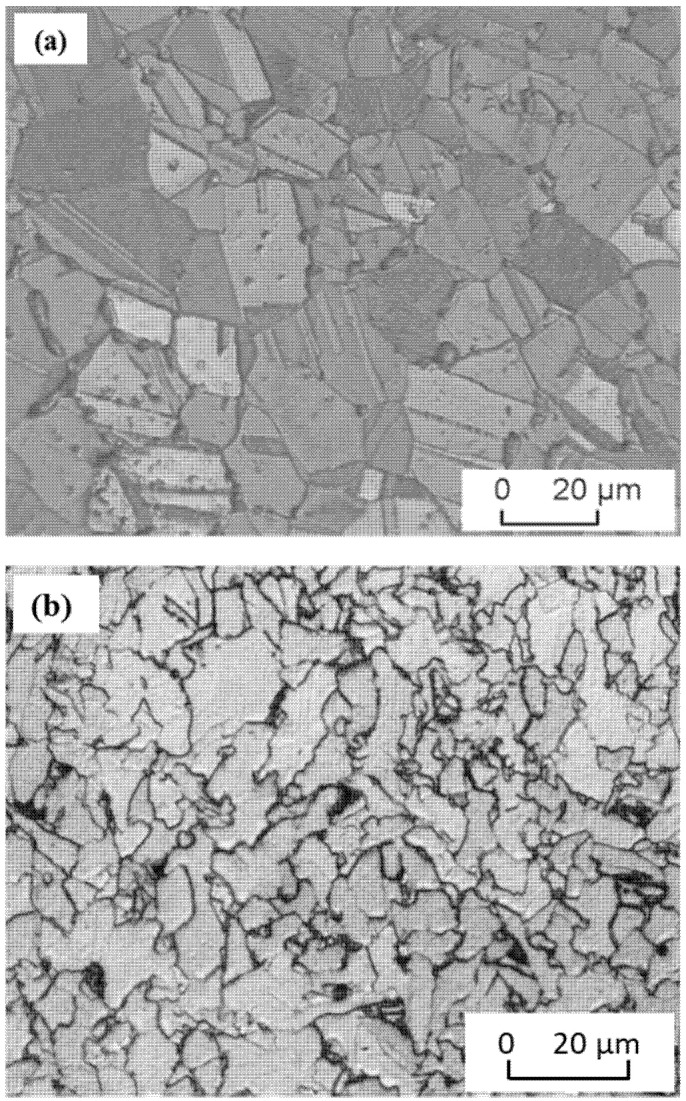
Optical microstructure of base materials used in this study. (**a**) Microstructure of brass (CuZn40) and (**b**) microstructure of low-carbon steel (steel 44).

**Figure 2:**
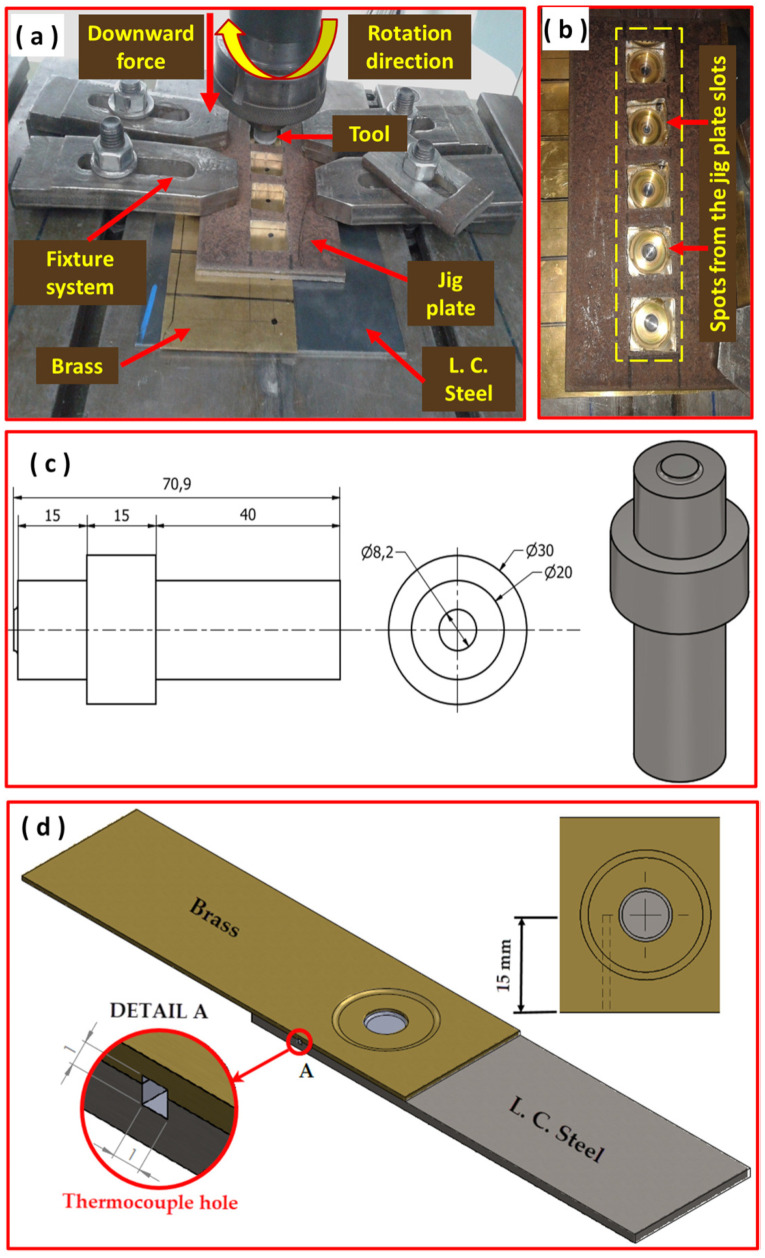
(**a**) FSSW process arrangement, (**b**) fixture plate supporting the sheets of spot joints, (**c**) graphical representation of the used FSSW tool arrangement, and (**d**) temperature measurement during FSSW (all dimensions in mm).

For microstructure investigation, the brass and steel samples were separately mechanically ground up to the final stage of SiC grit of 2400. (i) Brass was primarily polished using a solution of the following mixture: 260 mL oxide (Al_2_O_3_) suspension with file alumina particle (0.05 µm), 0.5 mL HF, 1 mL HNO_3_, and 40 mL H_2_O_2_. (ii) The samples were finally etched during the final polishing stage using Klemm III and potassium disulfide. The specimens were polished and etched as in steps (i) and (ii) for two or three times, until a scratch-free surface and the microstructure were revealed. Steel was etched after polishing in Nital 2% for 5 s. The microstructure of the starting brass and low-carbon steel is shown in Figure 1a,b, respectively. From the binary Cu–Zn system, brass with copper content less than about 63 wt.% is composed of a single α-phase [42]. With increasing the zinc content, more than 37 wt.% β-phase starts to appear. In the current study, brass with little dispersed β-phase can be distinguished due to the cold deformation of the alloy, as shown Figure 2a. Figure 2b shows the microstructure of the low-carbon steel used in this study. As can be seen from this micrograph, the microstructures are composed mainly of relatively large ferritic grains, while some smaller pearlite grains or dispersed pearlitic islands at the ferritic triple points of ferrite grains in small portions are spread across the whole area.

### 2.2. Friction Stir Spot Welding (FSSW)

The difficulty in fusion welding of the dissimilar metallic alloys, especially those are far different in their physical and mechanical properties, comes from the selection of the filler material that can match both alloys and also the high heat input that can severely deteriorate the properties of the welded area. Thus, the use of the solid state welding process can the best alternative to overcome these limitations. 

In this study, steel and brass sheets were cut into the dimensions of 100 mm × 200 mm. For the FSSW lap joint, the steel sheet was used as the lower sheet, and the brass sheet was used as the top sheet with an overlap of 40 mm. A fixture plate with rectangular slots of 30 mm × 30 mm slots was used to clamp the sheet and to support and stabilize the sheets during FSSW. Figure 2a shows the arrangement of the FSSW process, and the fixture plate is shown in Figure 2b. FSSW joints were conducted using a home-manufactured FSW machine with main specifications of load of 100 kN, Torque of 140 Nm, and rotation speed up to 3000 rpm existing in the FSW lab at Suez University, Egypt. FSSW tools made from tungsten carbide (WC) with a cylindrical probe (length: 0.9 mm, diameter: 8.2 mm) and shoulder diameter of 20 mm were used. There are many design considerations for manufacturing of FSW and FSSW tools [43,44,45]. However, the tool material was selected based on the tool material required for welding high-melting-point materials, as the work deals with both steel and copper alloys. Furthermore, a simple cylindrical tapered geometry design was used, as the preparation of complex features in the WC is quite difficult, and it will wear away very quickly.

Figure 2.c shows a graphical representation of the applied FSSW tool. The WC tool was fixed in a tool steel holder that made from H13 tool steel. After the welding process, the welded sheets were cut for further characterization. 

The FSSW joints were conducted at different rotation speeds of 1000, 1250, and 1500 rpm at a constant plunge rate of 0.1 mm/s, zero tilt angle, and distance controlled mode. The applied vertical load during welding was ranging between 11 to 14 kN. The temperature near to the stir zone was measured for FSSW experiments covering the applied number of rotations (83, 167, 250, 333, 500 R). A Modern Digital Multimeter (MDM) model UT61B, Zhejiang, China with thermocouple type “K” was used to measure the temperature. The thermocouple was inserted between the brass and steel sheets (as shown in Figure 2d), and the temperature values were recorded and plotted as a function of the time.

### 2.3. FSSW lap Joints Evaluation

**Figure 3:**
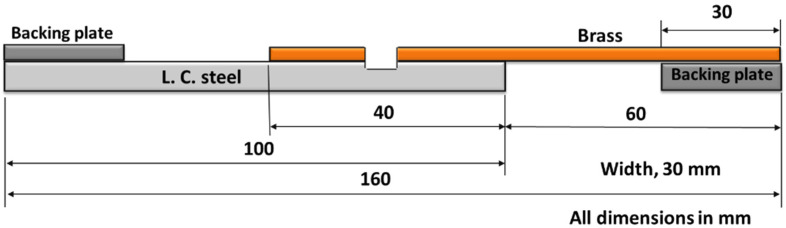
FSSW tensile shear test specimen with two backing plates to ensure axial loading of the specimens during the tensile shear test.

The starting sheets of copper and steel were cut into 100 mm × 200 mm. Copper was placed over steel with an overlap area of 40 mm × 200 mm. After welding, the whole dimensions of welded pair of materials were 160 mm × 200 mm, and it was cut into individual samples for investigations perpendicular to the length (200 mm); each is 30 mm in width and containing one spot, as shown in Figure 3. 

Lap shear tensile test was carried out on the FSSW joints with configuration of the sample, as shown in Figure 3, to measure the load-carrying capacity of the joints. The tensile test samples for the FSSW joints are of the width of 30 mm and a total length of 160 mm, with an overlap length of 40 mm, which are the same dimensions of the original welded plates. To ensure the axial loading of the test specimen, two packing pieces (30 mm × 30 mm) were adhesively joined on the weld specimens, as shown in Figure 3 [46]. A universal tensile testing machine, type Instron model 4210, (Norwood, MA, USA was used at crosshead speed of 0.05 mm/s. The load cells of the FSW and the tensile testing machine are calibrated by a specialized company; the same was performed periodically for the displacement measuring units.

The transverse cross section of the spot joints was investigated through macrographs and hardness measurements on a diagonal line through the joined materials. Low-load Vickers hardness tester HWDV-75, TTS Unlimited, Osaka, Japan was used with an indentation interspacing distance of 2 mm using a test load of 10 N and a dwell time of 15 s.

FEI Quanta FEG 250 Field Emission Gun Scanning Electron Microscope (FEGSEM), FEI company (Hillsboro, OR, USA), equipped with EDAX-OIM7 and controlled by TEAM software was used for microstructure investigation and elemental analysis of the joints. EDS line and point elemental analysis was carried out using a scan step size of 0.5 µm.

## 3. Results and Discussion

Many FSSW conditions were examined to explore the effect of the different parameters on developing high-quality dissimilar lap joints between brass (CuZn40) and low-carbon steel (St 44-2).

### 3.1. Heat Input in the FSSW Process

To determine the effect of the different welding parameters, the number of parameters has been reduced by combining the rotational speed (rpm) and the dwell time (s) into the number revolutions per joint as follows:

$$\mathrm{Total}\;\mathrm{number}\;\mathrm{of}\;\mathrm{revolutions}\;\left(R\right)=\;\mathrm{Tool}\;\mathrm{rotation}\;\mathrm{speed}\;\mathrm{per}\;\mathrm{second}\;\times \;\mathrm{dwell}\;\mathrm{time}\;\left(s\right)$$

Now, the rotational speed and the dwell time are reduced into one parameter, namely, the number of revolutions per spot (R). Moreover, the vertical load which represents the downward force (applied pressure) by the tool will be used to calculate the heat input in the welding process. Considering the friction force between the tool (pin surfaces and the pin shoulder) and considering an average friction coefficient (µ) equal to 0.5 [47,48,49] and using the applied vertical force (F), the rotational speed (ω) and the heat input (Q) are calculated as follows [50]:

(1) $$Q=\frac{13}{12}\;\mu \;\frac{F}{K_{A}}\;\omega \;r_{p}$$

where $r_{p}$ is the tool pin radius which equal to 4.1 mm and K_A_ is the value of shoulder contact surface area ($A_{C})$ divided by the total shoulder surface area (A) [50], which can be calculated as follows:

(2) $$K_{A}=\frac{A_{C}}{A}=\frac{\pi \;\left(r_{s}^{2}-r_{p}^{2}\right)}{\pi r_{s}^{2}}=\frac{\left(10^{2}-4.1^{2}\right)}{10^{2}}=0.8319$$

where r_s_ is the tool shoulder radius and r_p_ is the tool pin radius.

Basically, the angular speed $\omega \left(rad/min\right)=2\pi N\;\left(rpm\right)$ can be expressed in (rad/s) as follows:

(3) $$\omega \left(rad/s\right)=\frac{2\pi }{60}N\left(rps\right)$$

We can substitute the values of Equations (2) and (3) into Equation (1) and rewrite Equation (1) as follows:

(4) $$Q\left(J/s\right)=\frac{13}{12}*\;0.5*\;\frac{F}{0.8319}\;\frac{2\pi }{60}\;*0.0041\left(Nm/s\right)$$

Simply, heat input in the current case can be calculated as:

(5) $$Q\;\left(kJ/s\right)\;=2.794*10^{-7}\;\omega \;\left(rad/s\right)*\;F\;\left(N\right)$$

Equation (5) is valid and used for friction spot welding when multiplied by the linear travel speed. However, in FSSW, there is no travel speed but rotation in the same position for a dwell time, so that multiplying Q with dwell time $D_{t}$ (in s) gives the total generated heat (E) per spot weld, as described by Equation (6).

(6) $$E=Q\;*\;D_{t}\;\;\;\left(kJ\right)\;\;\;\;\;\;\;\;$$

**Figure 4:**
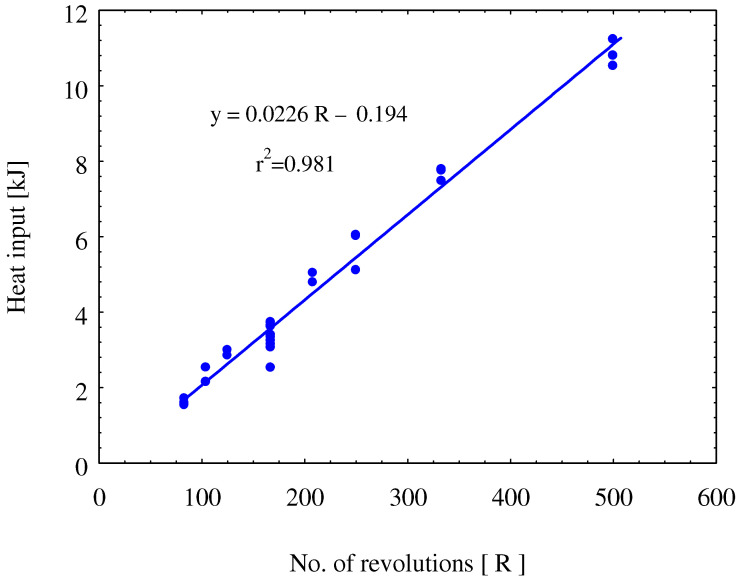
Linear fitting of the total heat input per spot weld as a function of the number of rotations on FSSW of low-carbon steel/brass (CuZn40) joints.

Figure 4 shows the effect of the total number of revolutions on the heat generated per spot weld. This figure shows the effect of the number of rotations as the key parameter in the heat generation upon FSSW process. Clearly it can be observed that increasing the number of rotations increases the heat input per spot. This will significantly affect the joints quality and microstructure. The heat input ranges from 11 kJ to 1.5 kJ based on the number of revolutions per spot.

Table 3 gives the calculated heat input at the different FSSW conditions, and Figure 4 shows the heat input against the number of revolutions. In Figure 4, the average total heat input has been well linear fitted with the total number of revolutions (R) per spot weld with a high coefficient of determination (r^2^ = 0.981). The linear equation describing the relation of the total generated heat per spot and the number of rotations is:

(7) $$Heat\;\left(kJ\right)=0.0226\;R-0.194$$

**Figure 5:**
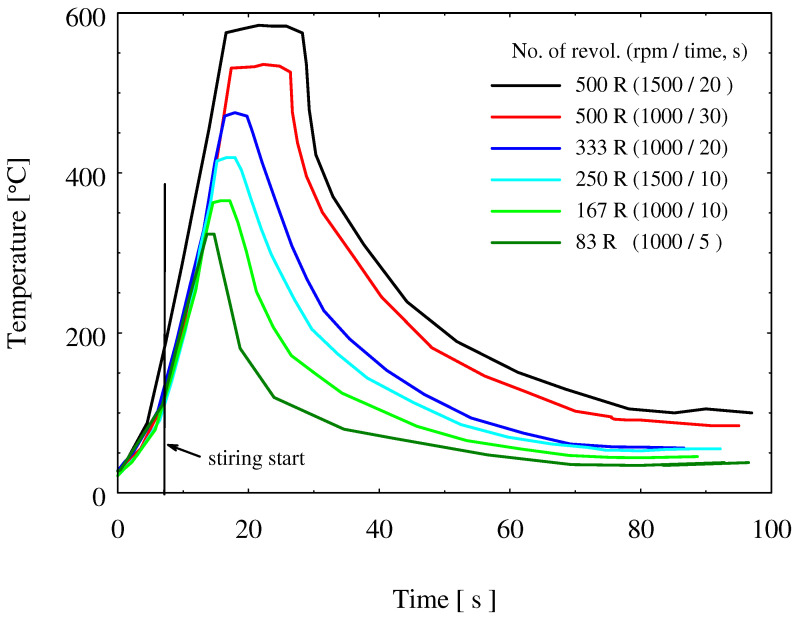
Temperature measured during some FSSW of low-carbon steel/brass joints.

Temperature was measured during the FSSW process for different experiments covering the applied number of revolutions range (83 to 500R) using different combinations of revolutions per minutes and dwell times. Figure 5 includes the curves of the measured temperature during the FSSW for some experiments. It is clear that the controlling parameter for heat input or temperature rise is not only the number of revolutions per spot but also the speed of the revolution. Practicing 500 revolutions within 20 s using a speed 1500 rpm is more effective than practicing the same number of revolutions in a time of 30 s using 1000 rpm. The first condition (1500 rpm, 20 s) raised the temperature to 583 °C, and a long time was needed to cool again, while a temperature of 535 °C was reached at the second condition (1000 rpm, 30 s). Comparing the experiments carried at the same speed of 1000 rpm for different dwell times of 5, 10, 20, and 30 s, it is obvious that the short dwell time is effective to raise the FSSW temperature, whereas the long dwell time is needed to generate heat at a rate higher than the heat dissipation rate through the different metallic units adjacent to the FSSW nuggets: base materials, jig plate, clamping units, FSSW tool, and the machine table. At 1000 rpm, the maximum attained temperature is 535 °C after 30 s; the maximum temperature falls to 323 °C at the shortest dwell time of 5 s (see Figure 5 and Table 4).

In Table 4, the maximum measured temperatures are listed for some FSSW experiments. Considering the melting of low-carbon steel (St 44-2) as 1539 °C and that for brass (CuZn40) as 900 °C [42], the relative absolute temperatures (T/T_m_) were calculated to show how far apart are the different welding temperatures from the recrystallization temperatures of the welded materials. It is obvious that the attained temperature during FSSW lies within the recrystallization temperature range of brass, while it is only at the boundaries or lower than the recrystallization temperature of steel.

### 3.2. Joint Quality and Macroscopic Investigation

**Figure 6:**
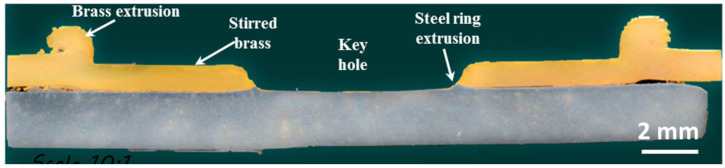
Macro image of an FSSW steel/brass joint showing the different zones using a total of 167 revolutions (rpm = 1000 and dwell time = 10 s).

Some samples were macroscopically investigated by a cut through the spot zone. The cut samples were molded to be easily handled during the metallographic preparation process. Due to the stirring occurred by the welding tool pin, a so-called keyhole is generated. Usually, this keyhole reaches the lower sheet of the joint, and the materials of the upper sheet are softened, pressed, and extruded to flow under the tool shoulder. With the applied force from the machine, the tool shoulder continues pressing the material removed by the pin, causing further extrusion of the softened brass to make it flow around the tool shoulder, producing a flash-like extruded brass, as shown in Figure 6.

Depending on the extent of the dwell time and the rotational speed, the tool pin may further press the underlying sheet (steel), causing little extrusion and producing a slight steel ring. Figure 6 also indicates that there is good cohesion between the brass and the undelaying steel sheet at the stirred region beneath the tool shoulder. This cohesion region is generated mainly by the heat generated from the friction process and the pressure practiced by the tool shoulder on the brass.

**Figure 7:**
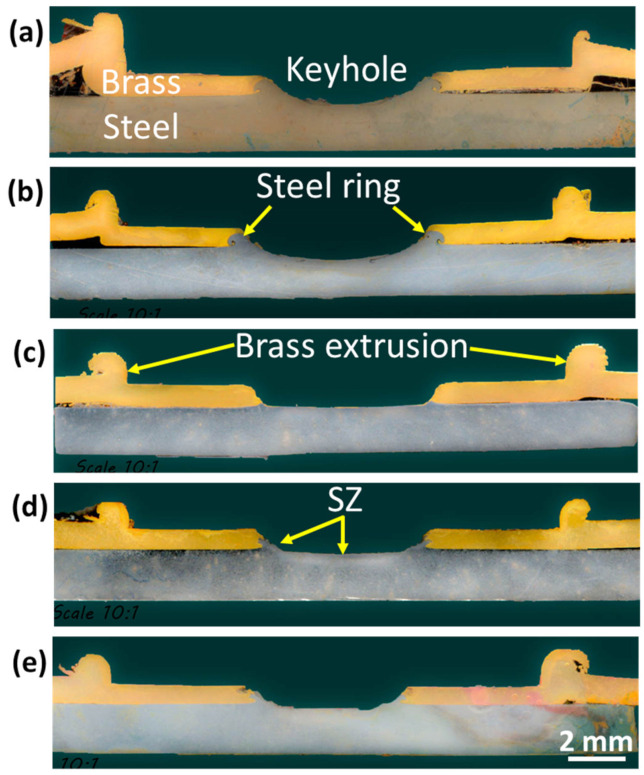
Macro images of FSSW steel/brass joints at different revolutions (R): (**a**) 83 R; (**b**) 167 R (penetration = 1.4 mm); (**c**) 167 R (1.8 mm); (**d**) 333 R; and (**e**) 500 R.

Figure 7 includes macroscopic images showing a comparison of the FSSW joint form at different welding conditions. Figure 7a shows the joint form at a low number of rotations (83 R) where the keyhole is still not well-formed. With increasing the number of rotations to 167 R, the weld nugget seems uniform around the keyhole (as shown in Figure 7b). Repeating the previous conditions while increasing the nominal downward displacement from 1.4 to 1.8 mm makes the keyhole deeper (Figure 7c) with more steel extrusion to the sides of the keyhole, and the cohesion of brass to steel appears with a lower area around the keyhole. Increasing the number of revolutions per spot to 333 and 500 increases the quality and the area of cohesion of brass to steel, as shown in Figure 7d,e, respectively. This is mainly due to the increased heat input for these two conditions, as shown in Figure 4 and Table 3.

### 3.3. Tensile Lap Shear Test Results

**Figure 8:**
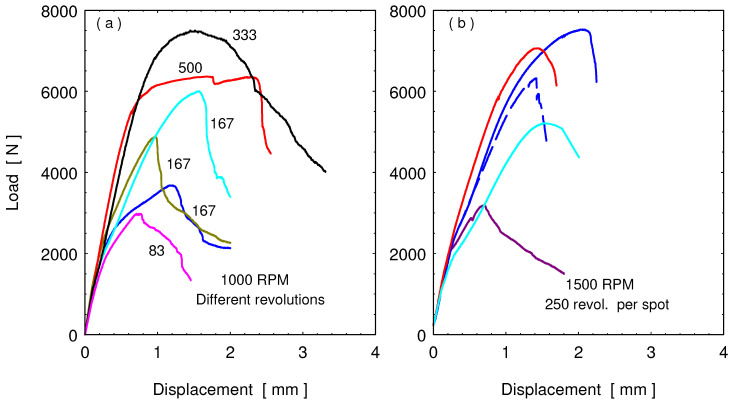
Load-displacement curves of joined samples at rotational speed of (**a**) at 1000 rpm and number of revolutions per spot (83, 167, 333, and 500 R) and (**b**) at 1500 rpm and 250 revolutions per spot.

**Figure 9:**
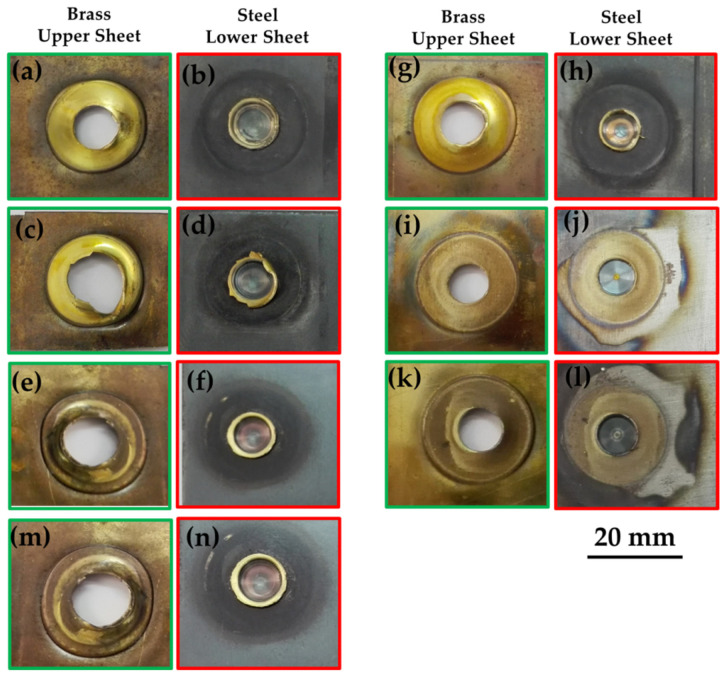
Photo images of the dissimilar fracture surfaces of the lap shear tensile tested FSSW steel/brass joints produced at different numbers of revolutions (R); (**a**,**b**) at 83 R, (**c**–**h**) at 167 R, (**i**,**j**) at 333 R, (**k**,**l**) at 500 R, and (**m**,**n**) at 250 R.

Tensile lap shear tests (TLST) were conducted on the FSSWed samples to evaluate the load-carrying capacity of the joints. The TLST results are presented in the load-displacement curves as shown in Figure 8. Figure 9 illustrates the fracture surface of lower surface of upper brass sheet and the upper surface of the lower steel sheet after the tensile test of the spot joints given in Figure 8. The lap shear tensile curves of the joints welded with the same rotation speed (1000 rpm) and different dwell times (5, 10, 20, and 30 s), which produced a different number of rotations per spot (from 83 to 500 revolutions), are shown in Figure 8a. This figure shows a variation of the tensile strength of the joint with the variation of the number of revolutions per spot. At the lower number of revolutions (83–167 R; Figure 9a–h), the displacement and the maximum force seem lower than that at the higher number of revolutions (333 and 500; Figure 9i–l) per spot. This is due to the improved joint quality in terms of good cohesion of brass on steel with the effect of increased total heat input per spot. The highest reached maximum tensile force at a rotational speed of 1000 rpm and the total number of rotations of 333 was 7.51 kN.

The FSSW of brass/steel welded at 250 R (1500 rpm and 10 s) is not reproducible as detected and represented in Figure 8b, where all displayed curves are for the same welding condition, and the resulting tensile data are very different. However, the maximum tensile force of 7.52 kN was attained at a rotation speed of 1500 rpm, or the total number of rotations per spot of 250. It can also be noticed from Figure 8a,b, that, with increased heat input (at the higher number of revolutions; 250, 333, and 500), the extent of displacement before fracture is higher than other conditions.

**Figure 10:**
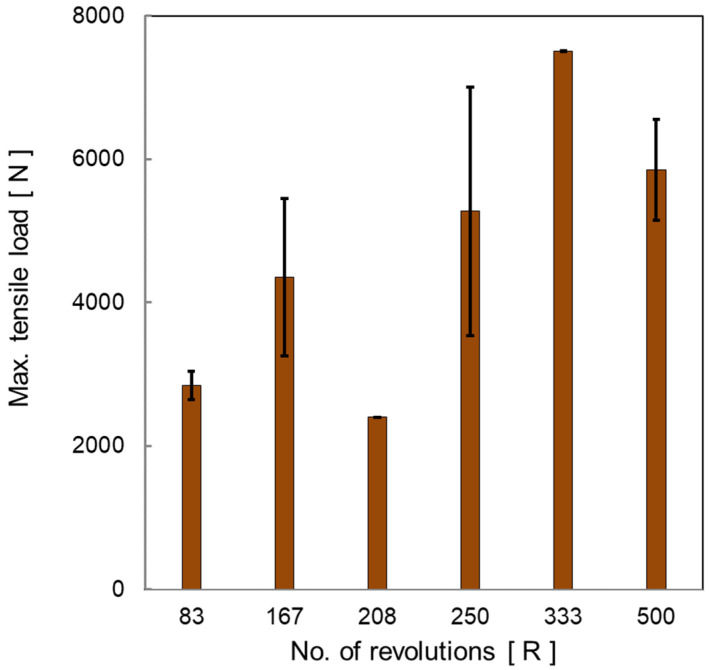
Average maximum tensile load of the tensile lap shear test of FSSW steel/brass joints manufactured by different revolutions per spot weld.

Table 3 includes the maximum tensile load for the different tested samples. The average of the maximum load carried by the joint is presented against the number of rotations per spot weld, as shown in Figure 10. It can be indicated that the maximum tensile increases with increasing the number of stirring rotations, which coincides with the highest total heat input per spot (Table 3). It can be noticed that there is a considerable error bar, which is calculated by the standard deviation of the obtained results for the same conditions. This means that the quality described by the maximum carried load of the joints produced by this manufacturing process is still not identical for the same condition, but there is a general trend showing increased load-carrying capacity of the joint with increasing the total generated heat during the process, which can be achieved by increasing the number of rotations per spot at considerable revolution speed in the range from 1000 to 1500 rpm.

To evaluate the success of such FSSW joints, the tensile shear results are compared with other works. According to the reported results by Abdullah and Hussein [51] for the FSSW of copper with carbon steel at plunge depths of 0.2 and 0.4 mm and different rotation speeds of 1120, 1400, and 1800 rpm, the tensile shear force of the welded joints was increased with an increase of the rotation speed and plunge depth, and the tensile shear force ranged between 510 N and 4.56 kN. The higher load-carrying capacities are attained mostly at a higher plunge depth using 1400 and 1800 rpm. The results from Figner et al. [52] show that FSSW of aluminum alloy AA5754 with galvanized steel using rotational speed ranging between 800 and 3200 rpm, dwell time range of 0 to 0.8 s, and plunge depth of 2.2 mm resulted in a maximum tensile lap shear force of 8.30 kN. However, the base materials of the joints of these results, or at least the softer component (aluminum alloy AA5754), have a higher strength than brass of the current work. The same can be mentioned for the joints [53] of AA5083, which were welded with steel alloy at various FSSW parameters. The shear forces of the joints with a maximum value of 4.02 kN was attained. Moreover, much lower shear force (3.20 kN) has been recorded by Sun et al. [20] for FSSW joints of AA6061-T6 with mild steel, which was achieved at a rotational speed of 700 rpm and a dwell time of 2 s. Mubiayi et al. [54] studied FSSW of pure copper (C11000) with the soft aluminum alloy AA1060; the higher load-carrying capacities of 5.23 kN and 4.84 kN were obtained at rotational speeds of 800 and 1200 rpm, respectively. A plunge depth of 1 mm was used for these successful cases. The base materials used by Mubiayi et al. [54] were softer than the current study materials. Based on the previously mentioned results and conditions, it could be concluded that the current attained results are comparable or even better among the achieved results from FSSW works.

### 3.4. Hardness Results of FSSW Joints

**Figure 11:**
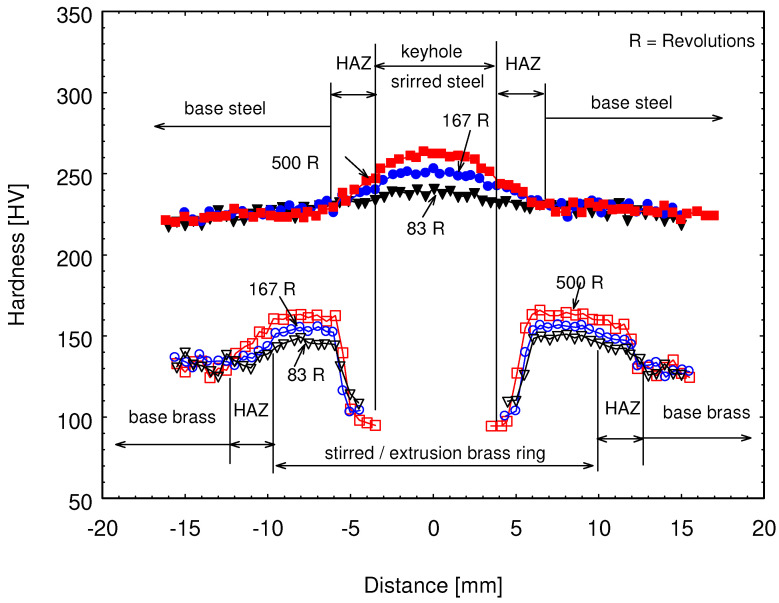
Vickers hardness profile measured on stirred brass and steel at the rotation speed of 1000 rpm for different dwell times, i.e., different revolutions (R) per spot (5 s: 83 R, 10 s: 167 R, and 30 s: 500 R).

Hardness measurements were carried out on the FSSW joint to explore the effect of stirring on the development of joint material strength. Figure 11 shows the hardness profiles measured on the joint sheet at different stirring dwell times, which produced different numbers of revolutions per joint. 

FSW or FSSW of materials with melting points very far apart such as steel/brass does not produce completely mixed dissimilar materials at the joint regions. Even if cohesion takes place, no wide range mixing occurs: materials of the top and bottom sheet stay distinguishable. Thus, hardness measurements were conducted on the welding regions of the two joined sheets individually. It seems that a small area of the steel sheet was affected, which is slightly bigger than the tool pin area. Beyond this area, the steel was not affected, and the hardness values of steel were scattered within a band around the base steel sheet hardness value (223 HV). At longer dwell time and higher number of rotations (500 R), the heat input was at a maximum, which provided a better condition for stirring the steel beneath the tool pin, leading to a clear increase of the hardness values reaching its maximum (262 HV), as shown in Figure 11 for steel in the middle zone. At the lower number of rotations (167 R), there was a little increase in the hardness value under the keyhole, reaching 248 HV, while a slight increase of the hardness above the hardness value of the base steel sheet was recorded a value of 238 HV on stirring for 83 R.

Brass containing 40 Zn has a melting temperature of around 900 °C. This means a temperature above 315 °C can lead to recrystallization of cold-worked brass. However, a much higher temperature (583 °C) was recorded on the FSSW of the brass/steel sheet (see Figure 5 and Table 4). Along with this high temperature, we also must keep in mind the mechanical stirring pressing and extrusion of brass, which is performed by the tool pin and shoulder. This gave us two oppositely working effects. The hardness measurements reflect the dominant effect on the strength of brass. Starting from the base brass sheet, the hardness increased with increasing the number of revolutions per spot. The highest hardness value of 163 HV was reached using 500 R at the stirred zone, while brass hardness using a number of rotations 83 R per spot showed a hardness value of about 149 HV. Both are located within the hardness ranges of the highest strength temper conditions (≥140 HV). At the highest temperature zone near the keyhole, brass began to recrystallize and became softer than the base cold-worked bass sheet. The lowest hardness value around 92 HV was recorded for the spot-welded test using 500 R, where the highest temperature was reached at the keyhole.

### 3.5. Microstructure, SEM, and EDS Analysis

**Figure 12:**
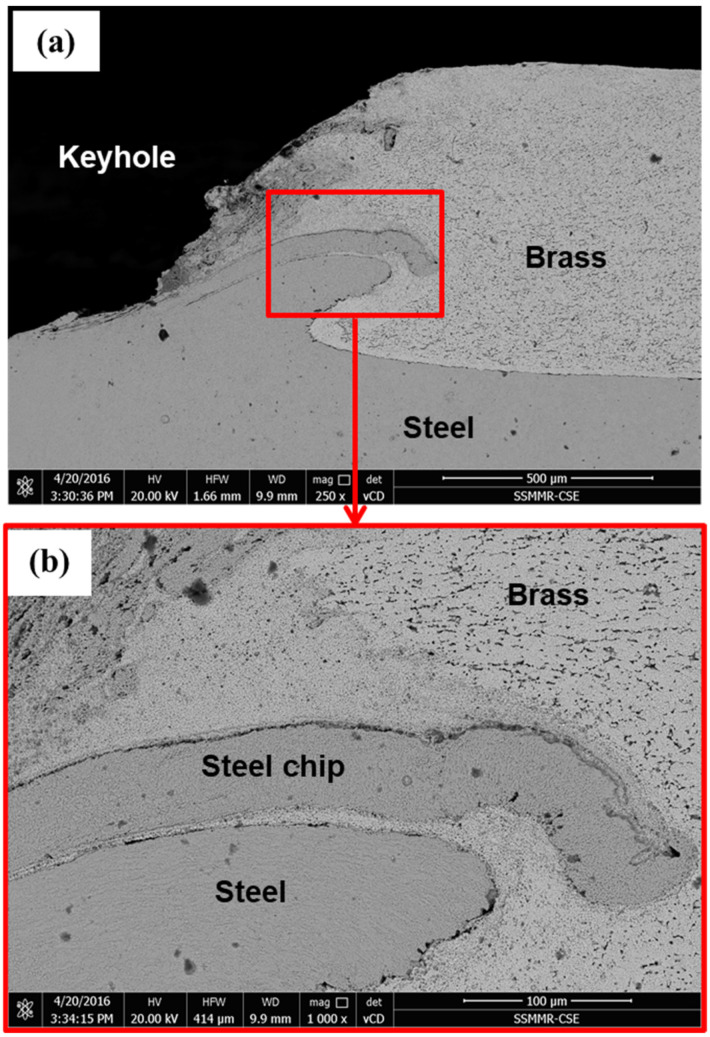
BSE SEM microstructure, right side of a cross-section through the keyhole of the joint produced by FSSW at 1000 rpm, dwell time of 20 s (333 R) (**a**) extrusion and flow of stirred steel into brass, and (**b**) higher magnification of the extruded steel region in an image (**a**).

Figure 12 shows the microstructure of the right side of the cross-section through the keyhole of the brass/steel joint produced by 333 rotations. Figure 12a shows good cohesion of brass to steel by the pressing force practiced by the tool shoulder at the generated heat and temperature, which could be around 472 °C (as indicated in Table 4). Frictional stirring of steel sheet by the bottom surface of the tool pin extruded some of the steel into brass. Penetration of the extruded steel into brass could increase the joint strength. The extruded steel appears in the form of a ring, as shown in the macro images shown in Figure 7, appears in the form of double rings, as shown in Figure 12a,b shows higher magnification of the region around the extruded steel where the brass is highly deformed and highly heat-affected by stirring and extrusion of both steel and brass. 

**Figure 13:**
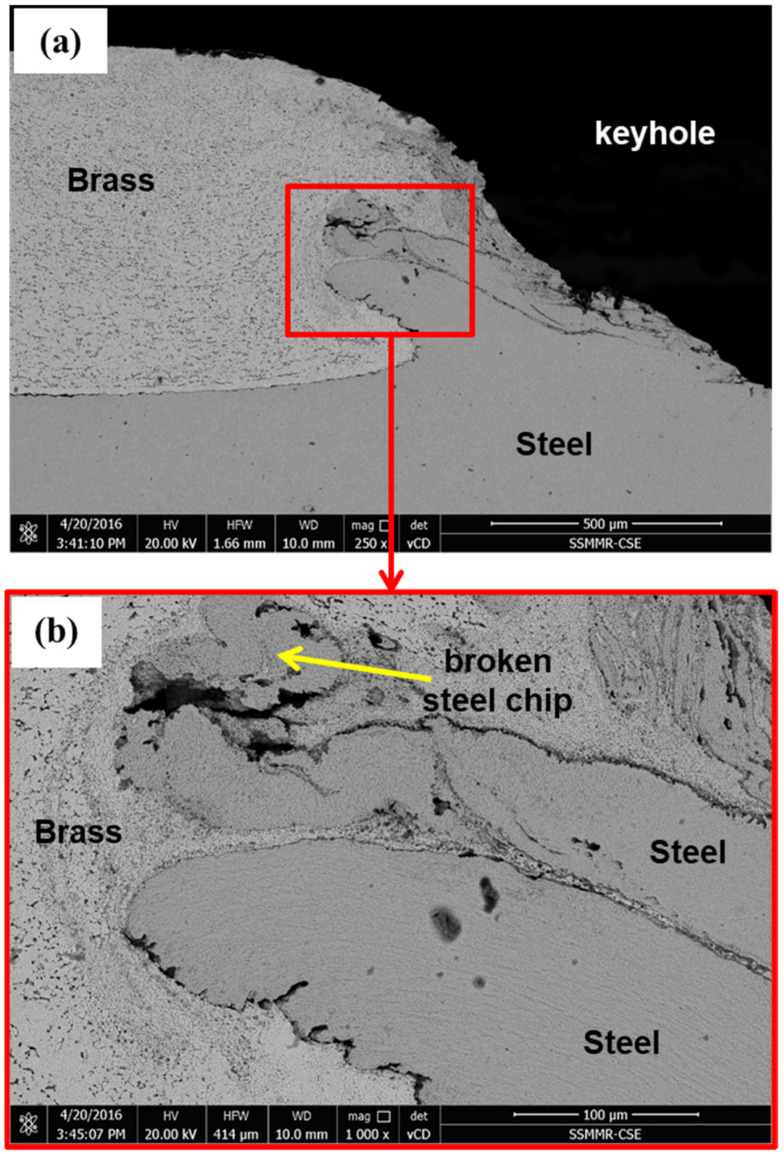
BSE SEM microstructure images, left side of a cross-section through the keyhole of the joint produced by FSSW at 1000 rpm, dwell time of 20 s (333 R). (**a**) Flow of steel extrusion into brass, and (**b**) higher magnification of the extruded steel in an image (**a**).

Microstructure investigation of the left side of the cross-section of the joint carried out at 1000 rpm and dwell time of 20 s (333 R) is shown in Figure 13. The general features explained in the right side of the joint in Figure 12 can also be seen in the microstructure of the left side of the joint shown in Figure 13, except the fracture of the steel chip extruded in the brass sheet. The upper steel extrusion chip seems thinner and especially longer than the lower one. This thinner chip faced resistance to flow in brass by the pressure from the tool shoulder; thus, this thin chip is bent and fractured. Near the keyhole, the temperature is at maximum due to the friction of the tool and the steel sheet. This high temperature facilitates the flow of steel extrusion into softened brass. Away from the keyhole, the heat sink increases, and the temperature steeply falls. This can be an additional resistance to the flow of the steel chip into brass. Although the fracture of steel extrusion can be considered as a discontinuity in the joint, it does not represent a welding defect as long as the area of cohesion of brass to steel is large enough. From the tensile lap shear test results (Figure 10 and Table 3), this welding condition (1000 rpm, 20 s, 333 R) produced a joint with the highest load-carrying capacity, where the average maximum load was 7505 N.

**Figure 14:**
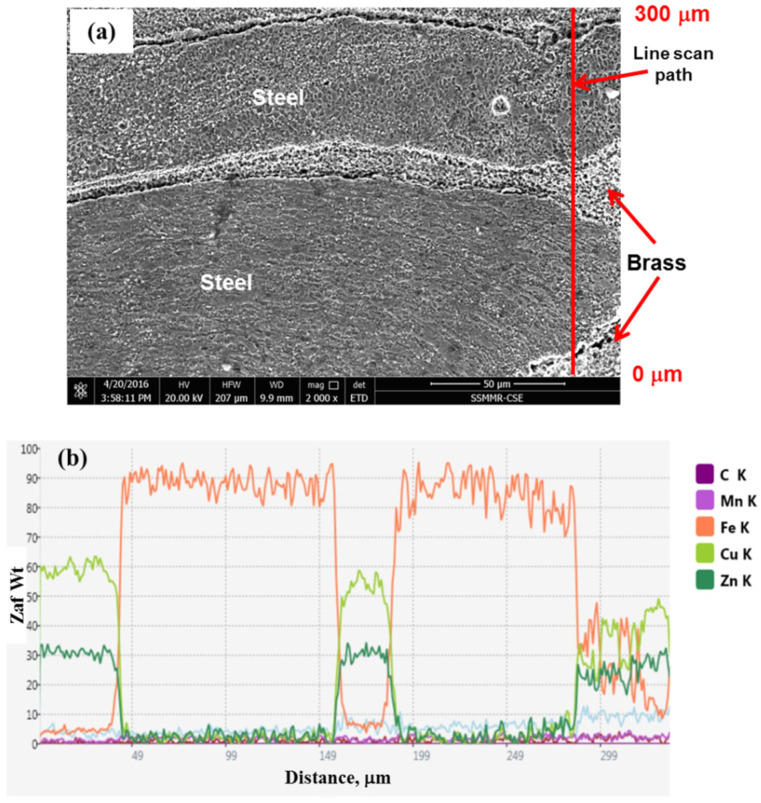
EDS analysis across the joint containing steel extrusion and brass for a region located on the right side of the cross-section through the FSSW joint by stirring at 1000 rpm, dwell time of 20 s (333 R). (**a**) EDS line path on SE SEM image, and (**b**) curves of the EDS elemental analysis.

EDS line scan was carried out on the sample welded at 1000 rpm for a dwell time of 20 s at the region containing steel and brass; see Figure 14. This image is a small area from the image shown in Figure 12. Normally, the analysis of steel is rich in iron, and the brass region is rich in copper and zinc. Let us concentrate on the transition length between steel and brass. There could be mutual atomic diffusion between steel and brass, which can be shown from light concentration gradient on the sound steel/brass interface. In friction stir welding of the brass/steel spot joint using a tool rotation of 1000 rpm and linear travel speeds of 250, 500, and 600 mm/min, Gao et al. [31,55] found that there is a mutual diffusion between iron and brass (Cu and Zn) over a length ranging between 70 and 120 nm. In the present work, the dwell time of 20 s using the same rotational speed (1000 rpm) offers a higher chance to introduce higher heat input in a localized spot. This increases the opportunity for mutual diffusion than that attained in FSW of brass/steel conducted by Gao et al. [31,55].

**Figure 15:**
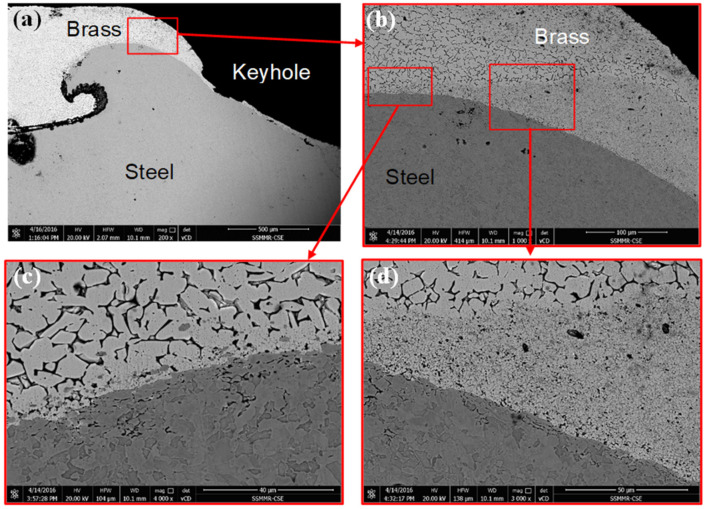
BSE SEM microstructure at different magnifications (**a**) 200×, (**b**) 1000×, (**c**) 4000×, (**d**) 3000× for the FSSW joints showing mechanical mixing and cohesion of brass on steel at the stirred of samples joined at a rotational speed of 1500 rpm, dwell time of 10 s (250R).

Figure 15 includes the microstructure of a region from the left side to the keyhole in a cross-section of the brass/steel spot joint produced at 1500 rpm for a dwell time of 10 s, which provides a total of 250 revolutions. Figure 15a shows the steel extrusion in the form of a hock inside brass. Excellent cohesion at the interface between brass and steel can be seen in Figure 15b. In further magnification, microstructural changes of brass can be seen, depending on the degree of deformation and heat input to the joint materials (Figure 15c,d).

**Figure 16:**
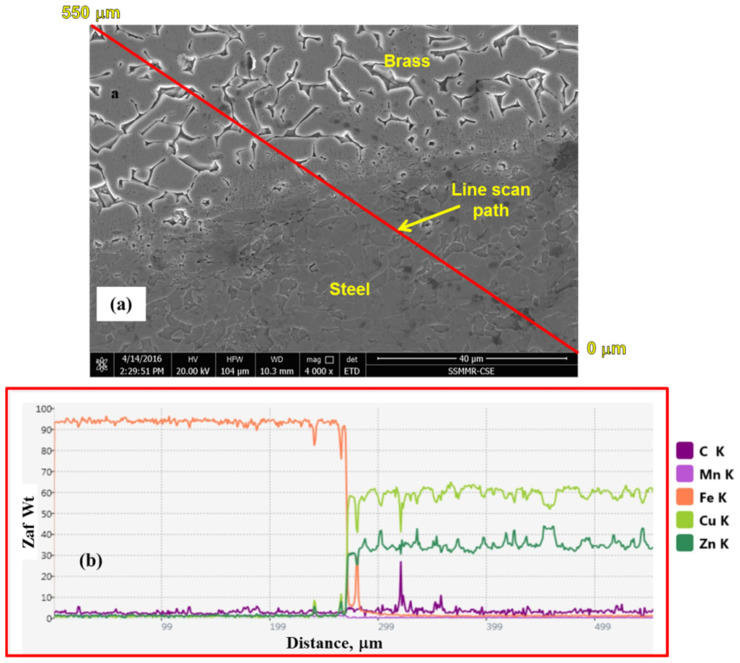
(**a**) EDS line scan from the lower right side to the upper left side of the SE SEM image of FSSW brass/steel joint by stirring at 1500 rpm and dwell time of 10 s (250 R), and (**b**) EDS line scan results.

Figure 16 shows the EDS line scan analysis of the brass/steel contacting region from the microstructure shown in Figure 15c. It can be noted that at the interface region between the brass and steel parts some traces of elements can be seen in both sides, which indicate the mutual diffusion of elements. Fe peak can be observed in the brass side as well as peaks of Cu and Zn in the steel side. This type of diffusion is expected to occur at this high temperature and high strain experienced during FSSW. Bonding of dissimilar metals during the different joining processes is explained on by many mechanisms [56,57,58]. In the current work, the attained results implied from Figure 12, Figure 13, Figure 14, Figure 15 and Figure 16 indicate that the main dominant bonding mechanism could be mechanical bonding and diffusion bonding on short scale at brass/steel interface.

**Figure 17:**
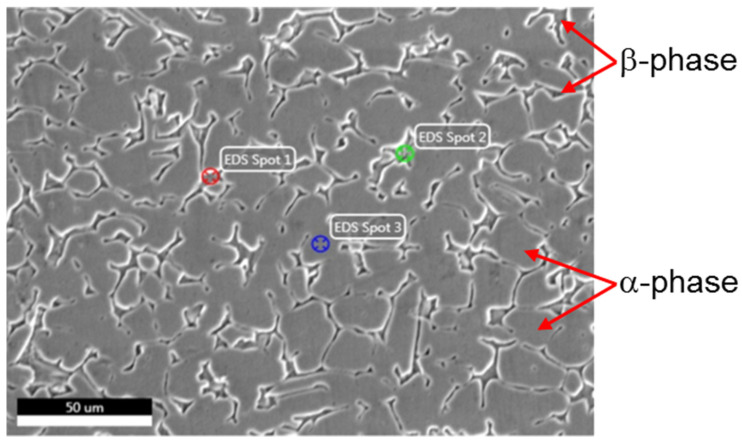
SE SEM microstructure, appearance of β-phase in the recrystallized structure of brass in the HAZ of samples joined at a rotational speed of 1500 rpm, dwell time of 10 s (250 R).

Table 5 includes the results of EDS analysis of different spots in the recrystallized brass, as shown in Figure 17. The elemental analysis shows that spots 1 and 2 are richer in zinc than spot 3 allocated in the matrix, representing α-brass phase, which has a zinc content of 32 wt.%, while the zinc content of spots 1 and 2 are 42.5 and 40.93 wt.%, respectively. The values of carbon that appeared in this analysis are mainly from contamination from the molding and sticking materials of the sample. 

In the next and final section, further investigation of the effect of FSSW conditions on the microstructure of brass are presented. Because brass represents the softer and weaker component of the joint, it determines the join’s strength; thus, that understanding its microstructural changes is of great importance. Figure 17 shows the microstructure of brass of the joint welded at 250 R (Figure 15) near the highest temperature region at the keyhole. It can be noticed that there are islands of β-phase distributed on discontinuous grain boundaries, while the matrix is mainly α-phase. This α-phase is dominating in brasses containing zinc content lower than 37 wt.%, according to the Cu–Zn phase diagram [42]. The β-phase appears in the as-received brass (Figure 1a) in the form of mechanical twins. At the regions adjacent to the high temperature, as shown in Figure 15, brass is recrystallized, and no more mechanical twins can be noticed, as shown in Figure 17.

### 3.6. General Discussion

The main contribution of this study is the investigation of the dissimilar spot joints between steel alloy and copper base alloy and the use of new parameters to justify the joint heat input and quality, which is the tool revolutions per spot as one parameter encompasses both the tool rotation rate and dwell time. In this section, some normalized points from the applied process conditions and the results to be applied for other FSSW are presented. In this work, three tool rotation speeds (1000, 1250, and 1500 rpm) and four dwell times (5, 10, 20, and 30 s), which form 12 process conditions, were reduced to seven rotations per spot (83, 104, 125, 167 250, 333, 500 R). The studied materials have melting points very far apart: brass (CuZn40) having a melting point of 900 °C and the relatively harder material represented by low-carbon steel with a much higher melting point (1530 °C). The normalized measured temperature at the applied number of revolutions (Table 4) show that the welding conditions resulted in generating heat, which raised the temperature up to ≈0.4 T_m_ for such steel and ≈0.6 T_m_ for such soft brass, which could be suitable conditions for such material joining. Furthermore, the generated heat at a number of revolutions of 250 or higher (Table 3 and Figure 4) was also a reasonable level to produce a dissimilar joint with good load-carrying capacity of 4 to 7.5 kN, as shown in Figure 10. Whenever such relative temperature is reached, a successful FSSW joint can be achieved for similar combinations of materials with very different melting points. Even the rotational speed and the dwell times were normalized into the number of revolutions per spot, keeping in mind that the rotational speed and the dwell time should be high enough to provide enough higher rate of heat generation. If other FSSW tools are used, the generated heat input by the new tool alone would be estimated using the given equations to see if the heat input value is enough to place the selected new tool geometry or the new process conditions (rotational speed and dwell time) in the heat input range to produce acceptable load-carrying capacity. The new tool geometry (such as pin height) should, of course, be related to thickness of the top sheet materials thickness; here, it was 0.1 mm shorter than the top sheet materials thickness. Heat input calculation for the FSSW of steel–brass was found to be linearly proportional with the number of revolutions per spot joint. Maximum heat input obtained was 11 KJ at the number of revolutions of 500. The temperature measurement during FSSW showed agreement with the heat input dependence on the number of revolutions. However, at the same number of revolutions of 500, it was found that the higher rotation speed of 1500 rpm resulted in higher temperature of 583 °C compared to 535 °C at a rotation speed of 1000 rpm. This implies the significant effect for the rotation speed in the increase of temperature. The macro investigations of the friction stir spot-welded joints transverse sections showed sound joints at the different investigated parameters with significant joint ligament between the steel and brass. The tensile shear load results showed wide scattering at the same value of number of revolution, with maximum tensile shear load of about 7.52 kN attained at a rotation speed of 1500 rpm and 10 s, or the total number of rotations per spot of 250. 

Tensile test results are usually scattered in a narrow range. The scattering of TLST results could not be compared with the scattering of the fusion welded or even with the FSW samples. The variation of the TSLT results shown in Figure 10 could be related to variation in behavior of the stirred at the keyhole and the form of extrusion of steel chips and brass around the keyhole. Figure 12, Figure 13, Figure 14, Figure 15 and Figure 16 show the different forms of cohesion at the steel–brass interface, which results in different load-carrying capacity of the samples. However, sticking to the conditions producing a high heat input range (i.e., at 250 revolutions per spot or higher) produces lap joints with relatively high load-carrying capacity.

The hardness measurement across the transverse section of the spot joint showed an increase in the carbon steel (lower sheet) with a maximum of 248 HV and an increase of brass hardness at the mixed interface between brass and steel and significant reduction in the stir zone. Microstructural investigation of the joint zone showed mechanical mixing between steel and brass, with the steel extruded from the lower sheet into the upper brass sheet. The EDS analysis across the interface showed a concentration gradient for both Fe into brass and Cu into steel, which implies the mutual diffusion at the experienced thermomechanical conditions. The current study showed the importance of parameters optimization using the different optimization software to minimize the number of experiments. Thus, the results of this study can used in an optimization study using Taguchi method, for example. Furthermore, the refill FSSW technology can be used to improve the joint appearance and quality. 

## 4. Conclusions

Based on the obtained results and analysis, the following conclusions can be outlined:

(1) Whenever a generated FSSW heat over 4 kN is secured by any parameters combination, successful joints can be achieved.
(2) Heat input for the FSSW of steel–brass was found to be linearly proportional with the number of revolutions per spot joint, and the maximum heat input obtained is 11 kJ at the number of revolutions of 500.
(3) Application of the acceptable range of FSSW revolution per spot heats the joint zone to a normalized temperature of ≈0.4 T_m_ for steel and ≈0.6 T_m_ for brass and produces good steel/brass adhesion, which is strengthened by the mutual atomic diffusion.
(4) Considering other successful FSSW conditions, an appropriate tool geometry with a height of 0.1 mm less than the top sheet thickness produces good joint load-carrying capacity.
(5) FSSW of steel/brass joints with a number of revolutions ranging between 250 to 500 revolutions per spot at appropriate tool speed range (1000–1500 rpm) produces joints with high load-carrying capacity from 4 kN to 7.5 kN.

## Figures and Tables

**Table 1:** The chemical composition of low-carbon steel (St 44-2) and brass (CuZn40) sheets (in Wt.%).

Element (Wt. %)	C	Si	Mn	P	S	Cr	Ni	V	Nb	Cu	Zn	Pb
St 44-2	0.052	0.034	0.875	0.01	0.003	0.017	0.05	0.036	0.026	0.03	-	0.005
CuZn40	-	0.002	0.005	0.007	-	-	-	-	-	61.3	38.6	0.025

**Table 2:** The mechanical properties of the starting sheet materials: low-carbon steel (steel 44-2) and brass (CuZn40).

Material	Properties			
	Yield Stress σ_y_ (MPa)	Tensile Strength σ_uts_ (MPa)	Fracture Strain ε_Fr_ (%)	Hardness HV
Steel 44-2	461	525	19.4	223 ± 2.4
CuZn40	250	434	40	128 ± 2.3

**Table 3:** Welding conditions, the generated heat input, and the tensile lap shear maximum force carried by the joint.

Max. Tensile	Energy	Power	No. of	Machine	Dwell	Rotation	Joint
Load			Rotations	Load	Time	Speed	Numbering
(kN)	(kJ)	(kJ/s)	(R)	(kN)	(s)	(rpm)	
2.7	1.7	0.34	83	11.7	5	1000	1
3	1.6	0.32	83	10.96	5	1000	2
-- *	1.6	0.31	83	10.7	5	1000	3
--	1.5	0.31	83	10.5	5	1000	4
--	2.5	0.51	104	13.8	5	1250	5
--	2.1	0.43	104	11.7	5	1250	6
1.3 **	3	0.6	125	13.6	5	1500	7
--	2.9	0.6	125	13	5	1500	8
3.6	3.3	0.33	167	11.4	10	1000	9
3.3	3.7	0.37	167	12.8	10	1000	10
--	3.2	0.32	167	11.1	10	1000	11
6	2.5	0.25	167	8.6	10	1000	12
4.9	3.2	0.32	167	10.7	10	1000	13
--	3.1	0.31	167	10.5	10	1000	14
4	3.4	0.34	167	11.6	10	1000	15
1.5	3.6	0.36	167	12.4	10	1000	16
--	3.7	0.37	167	12.5	10	1000	17
2.4	4.8	0.48	208	13.1	10	1250	18
--	5	0.5	208	13.8	10	1250	19
3.2	5.1	0.51	250	11.6	10	1500	20
5.2	6	0.6	250	13.7	10	1500	21
2	6	0.6	250	13.8	10	1500	22
3.8	NA	NA	250	NA	10	1500	23
1.6	NA	NA	250	NA	10	1500	24
7.5	NA	NA	250	NA	10	1500	25
7.1	NA	NA	250	NA	10	1500	26
6.3	NA	NA	250	NA	10	1500	27
7.5	7.8	0.39	333	1.3	20	1000	28
7.5	7.5	0.37	333	12.8	20	1000	29
--	7.8	0.4	333	13.3	20	1000	30
6.4	11.2	0.37	500	12.8	30	1000	31
5.4	10.8	0.36	500	12.3	30	1000	32
--	10.5	0.35	500	12	30	1000	33

* The joints that show “--” in the “Max. tensile load” have been consumed in investigations other than tensile test such as hardness testing and macro- and microstructure investigation. ** FSSW joints with a maximum load lower than 2 kN have been considered as failed joints.

**Table 4:** Measured temperature during brass/steel FSSW experiments related to the absolute melting temperature of the base materials.

Rotation Speed, (rpm)	Time (s)	No. of Rotations, (R)	Maximum Temperature, (°C)	T/T_m_ (Steel)	T/T_m_ (Brass)
1500	20	500	583	0.47	0.73
1000	30	500	535	0.45	0.69
1000	20	333	472	0.41	0.64
1500	10	250	420	0.38	0.59
1000	10	167	363	0.35	0.54
1000	5	83	323	0.33	0.51

**Table 5:** EDS elemental analysis of spots 1 and 2 (β-phase) and spot 3 (α-phase).

Element	(wt.%) Spot 1	(wt.%) Spot 2	(wt.%) Spot 3
Fe	0.6	1.6	0.8
Cu	54.1	53.2	63.3
Zn	42.5	40.9	32.1

## Data Availability

The data presented in this study are available on request from the corresponding author. The data are not publicly available due to the extremely large size.
